# Retinal Ganglion Cells and Circadian Rhythms in Alzheimer’s Disease, Parkinson’s Disease, and Beyond

**DOI:** 10.3389/fneur.2017.00162

**Published:** 2017-05-04

**Authors:** Chiara La Morgia, Fred N. Ross-Cisneros, Alfredo A. Sadun, Valerio Carelli

**Affiliations:** ^1^IRCCS Institute of Neurological Sciences of Bologna, Bellaria Hospital, Bologna, Italy; ^2^Neurology Unit, Department of Biomedical and Neuromotor Sciences, University of Bologna, Bologna, Italy; ^3^Doheny Eye Institute, Los Angeles, CA, USA; ^4^Department of Ophthalmology, David Geffen School of Medicine at University of California Los Angeles, Los Angeles, CA, USA

**Keywords:** optic nerve, retinal ganglion cells, melanopsin, circadian rhythms, Parkinson’s disease, Alzheimer’s disease, Huntington’s disease

## Abstract

There is increasing awareness on the role played by circadian rhythm abnormalities in neurodegenerative disorders such as Alzheimer’s disease (AD) and Parkinson’s disease (PD). The characterization of the circadian dysfunction parallels the mounting evidence that the hallmarks of neurodegeneration also affect the retina and frequently lead to loss of retinal ganglion cells (RGCs) and to different degrees of optic neuropathy. In the RGC population, there is the subgroup of cells intrinsically photosensitive and expressing the photopigment melanopsin [melanopsin-containing retinal ganglion cells (mRGCs)], which are now well known to drive the entrainment of circadian rhythms to the light–dark cycles. Thus, the correlation between the pathological changes affecting the retina and mRGCs with the circadian imbalance in these neurodegenerative diseases is now clearly emerging, pointing to the possibility that these patients might be amenable to and benefit from light therapy. Currently, this connection is better established for AD and PD, but the same scenario may apply to other neurodegenerative disorders, such as Huntington’s disease. This review highlights similarities and differences in the retinal/circadian rhythm axis in these neurodegenerative diseases posing a working frame for future studies.

## Introduction

Alzheimer’s disease (AD) and Parkinson’s disease (PD) are the most frequent age-related neurodegenerative disorders with an increasing prevalence with age (1, 2). They are both characterized by the frequent occurrence of sleep problems and circadian rhythm dysfunction (3–6). In the last decade, the role of the eye in influencing and regulating circadian rhythms has been clarified, starting from the discovery of the intrinsically photosensitive melanopsin-containing retinal ganglion cells (mRGCs) (7, 8). These cells constitute a small subset of regular retinal ganglion cells (RGCs) consisting of about 1–2% of the total, and they give origin to the retinohypothalamic tract through which they project to the suprachiasmatic nucleus (SCN) of the hypothalamus synchronizing circadian rhythms to the light–dark cycle (9). Besides this predominant function, they also play an important role in many non-visual functions of the eye, regulating sleep through the connections with the ventrolateral preoptic nucleus (VLPO) and the lateral hypothalamus (LH), melatonin secretion, and its suppression through the connections with the pineal gland, pupillary reflex through the olivary pretectal nucleus, and also visual functions through the projection to the lateral geniculate nucleus (10–12).

In this review, the likely influence of the mRGC system in the pathogenesis of circadian misalignment in AD and PD is discussed, highlighting similarities and differences, starting from the observation that in both diseases, loss of regular RGCs has been documented by both histological and optical coherence tomography (OCT) studies, thus suggesting that the retina is actively and primarily involved in the neurodegenerative process characterizing both disorders. In fact, many studies describe optic neuropathies associated with AD and PD, which, however, display different patterns of RGC and axonal loss, possibly reflecting different pathogenic mechanisms. We here explore the connection between the eye and circadian functions and dysfunctions in AD and PD with particular reference to the mRGC system and its contribution to circadian functions.

## Evidence of Inner Retina Pathology in AD and PD

### Alzheimer’s Disease

Histological and OCT studies in AD demonstrated a significant loss of RGCs and consequent axonal depletion in the optic nerve. Hinton and colleagues in 1986 reported the first histological demonstration of optic neuropathy in AD describing loss of RGCs and axons in the optic nerve (13). After this seminal work, other histological studies reported degeneration of the inner retina in AD, more pronounced in the superior and inferior sectors of the optic nerve (14–20).

**Figure 1:**
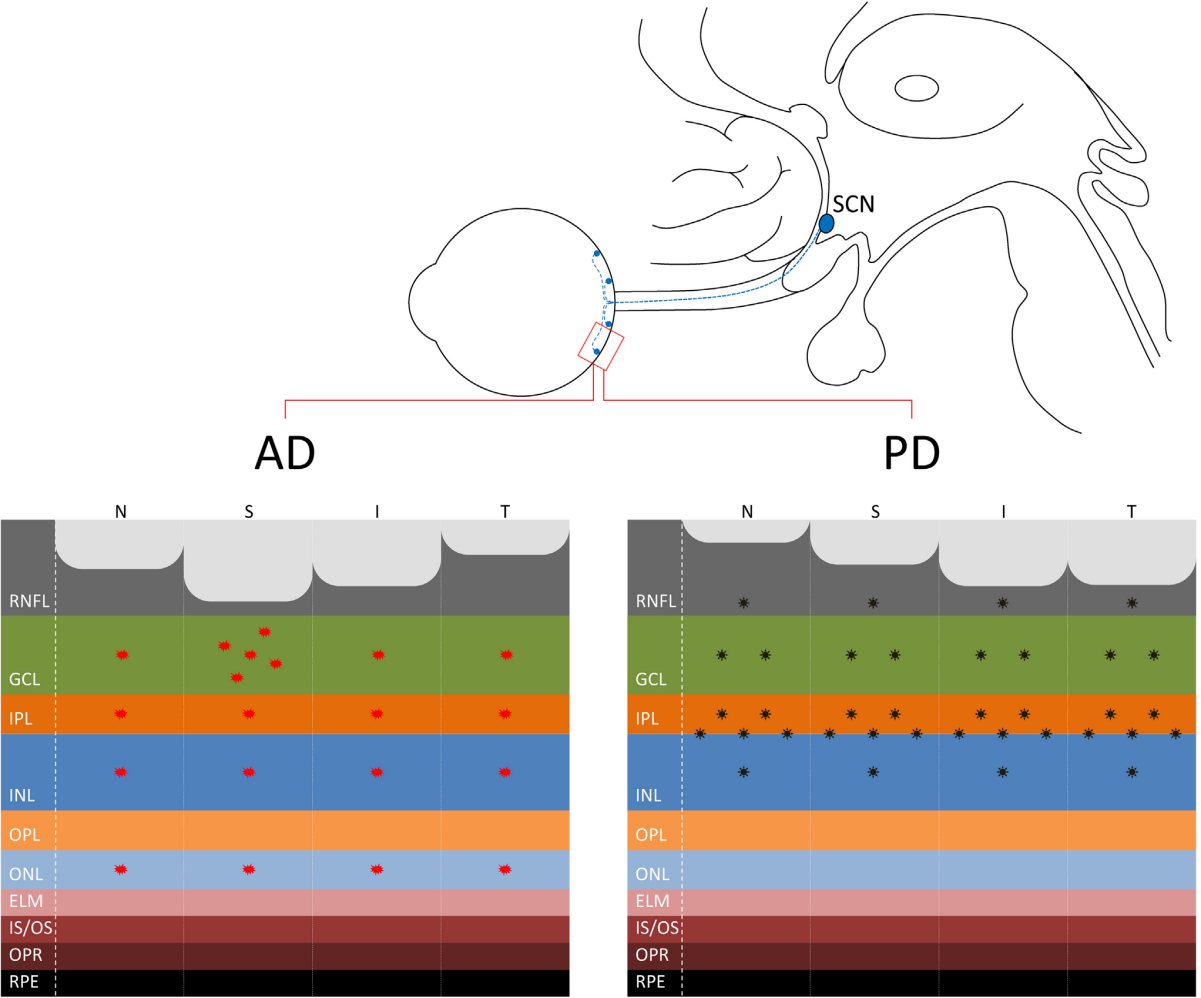
**(Upper panel)** The connection between the eye and the suprachiasmatic nucleus (SCN) of the hypothalamus through the retinohypothalamic tract, originating in the retina from melanopsin-containing retinal ganglion cells (mRGCs) (in blue), is shown. **(Lower panel)** At the retina level, where are located also the mRGCs, the distinct pattern of axonal loss [retinal nerve fiber layer (RNFL) thinning] demonstrated by optical coherence tomography studies is reported for Alzheimer’s disease (AD) (21) with a more pronounced loss in the superior quadrant (left) and Parkinson’s disease (PD) with a more evident loss in the infero-temporal quadrants of the optic nerve (44, 46, 49) (right). Moreover, the figure depicts the pattern of β-amyloid deposition in AD, more evident in the superior quadrant and ganglion cell layer (GCL) [for a review, see Ref. (30)], and α-synuclein in PD in the inner retina and in particular at the inner plexiform layer (IPL)–inner nuclear layer (INL) interface (51, 52).

These histological findings are corroborated by many recent OCT studies pointing to retinal nerve fiber layer (RNFL) thinning in AD, as confirmed by a recent meta-analysis of 11 OCT studies in AD (21). RNFL thinning is more pronounced in the superior sector of the optic nerve (20–23) and is age related (20) (Figure 1). This pattern of RGC loss is consistent with the predominant inferior visual field defect described in AD patients (24). Moreover, a recent OCT study using segmentation analysis in a large series of AD patients showed a significantly reduced macular retinal ganglion cell–inner plexiform layer thickness in AD retinas compared to controls (25).

The pattern of axonal loss in the optic nerve, for example, the prominent superior quadrant involvement, is consistent with the histological findings that magnocellular RGCs are more vulnerable to AD pathology (16), which also resembles the pattern of RGC loss described in glaucoma (26). Recently developed *in vivo* imaging methods, such as the detection of retinal cells undergoing apoptosis (DARC), are extremely promising in quantifying and visualizing *in vivo* RGC loss in AD retinas (27).

The presence of the cerebral hallmarks of AD, such as amyloid plaques, in the retina gives strength to the specific vulnerability of the eye, and in particular of the inner retina to AD pathology. Koronyo-Hamaoui and colleagues provided the first demonstration of extra-cerebral Aβ deposits in postmortem human flat-mounted retinas of AD patients and *ex vivo* in APPSWE/PS1ΔE9 transgenic mice after curcumin administration (28). Subsequent studies confirmed the presence of specific amyloid pathology, including both extracellular plaques and intracellular Aβ deposits, more evident in the superior quadrant, and increased Aβ peptides levels in human AD retinas (20, 29, 30) (Figure 1). Other promising imaging techniques, recently developed for visualizing amyloid deposits in AD retinas, include hyperspectral imaging (31), the use of cross-polarizers (32), and the polarization-sensitive OCT (33). Schön and coauthors also demonstrated the presence of the other hallmark of AD pathology, such as the phosphorylated tau, in human AD retinas (34).

Finally, our group recently demonstrated that a specific subpopulation of RGCs, the mRGCs, are specifically lost in AD and affected by the amyloid pathology. In fact, using melanopsin and Aβ co-staining, our group showed that Aβ deposits are evident within and around these cells affecting also mRGC neuritis (20). Remarkably, the loss of these cells is evident even with a normal RGC count, pointing to a specific AD pathology affecting mRGCs (20). The loss of these cells is particularly relevant for interpreting the occurrence of sleep and circadian disturbances in AD (see next section).

### Parkinson’s Disease

The occurrence of visual problems is a frequent finding in PD patients. These include blink, dry eyes, reduced visual acuity, contrast sensitivity, color vision abnormalities, oculomotor disturbances, and visual hallucinations (35, 36).

In particular, contrast sensitivity abnormalities are related to dopamine depletion at the retina levels (37–39) and can be partially reversed by the administration of l-DOPA therapy in PD patients (40). In fact, dopaminergic amacrine cells in the retina regulate the center-surround organization of RGC receptive fields and their dysfunction leads the retina to be in an inappropriately dark-adapted state (35). Color vision in PD patients is an early sign involving, at difference with the color defects observed with aging, the protan–deutan axis (red–green) (41). Interestingly, color vision abnormalities have good discriminative power in distinguishing PD patients from controls in the early stage of disease and may predict the conversion of idiopathic REM behavior disorder patients to PD (42, 43). However, the Farnsworth–Munsell 100 Hue test, commonly used for testing color abilities in PD, is influenced also by cognitive functions such as executive functions, and this must be taken into account in the interpretation of these results.

Besides the retinal dopaminergic depletion, which explains the occurrence of contrast sensitivity abnormalities in PD, there are multiple evidences pointing to RGC loss in PD (44, 45) (Figure 1). The presence of optic neuropathy has been reported by many OCT studies and, interestingly, the pattern of axonal loss resembles that typically seen in mitochondrial optic neuropathies, affecting the temporal sector of the optic nerve, i.e., the papillomacular bundle (RGC) (44, 46, 47). This pattern of RGC loss, which affects predominantly the parvocellular component, is clearly distinguishable from that described in AD, for which more frequently the magnocellular RGCs are affected (16, 20, 21) and other Parkinsonian syndromes, such as multiple system atrophy (Figure 1) (48).

Moreover, the optic neuropathy in PD is more pronounced in the eye contralateral to the most affected body side (46), suggesting a congruent asymmetry of the neurodegenerative process affecting also the retina. This asymmetry has been also documented for the foveal remodeling demonstrated in PD patients, as a hallmark of retinal pathology in PD (49, 50).

Finally, recent studies reported the presence of α-synuclein deposition in the retina of PD patients and in particular in the inner retina, pointing to a specific PD pathology affecting the eye (51, 52) (Figure 1). Interestingly, the staining of phosphorylated synuclein (p-syn) shown by Beach and colleagues (52) affected a large cell with an extensive dendritic tree, which resembles an mRGC. The possible occurrence of α-synuclein pathology affecting the mRGCs in PD may contribute to the occurrence of circadian dysfunction in PD that remains to be tested.

## Circadian Rhythm Dysfunction in AD and PD

### Alzheimer’s Disease

Sleep and circadian disturbances are a frequent complaint of AD patients, appearing in the majority of cases early in the disease course and including daytime somnolence, increased sleep latency, and frequent night-time awakenings with sleep fragmentation (3). Some of the sleep changes described in AD are the same reported with aging, such as the reduction of slow wave sleep and the difficulty in sleep maintenance (53). Other sleep abnormalities, such as the reduction of REM sleep, are more specifically related to AD (54).

Circadian rhythm abnormalities are reported in AD patients even in the early stage of the disease including a delayed phase of the temperature rhythm (55), sundowning, i.e., the appearance of behavioral agitation in the evening (56), the reduction of melatonin levels during the night (57), and the circadian expression profile of clock genes (57, 58). Moreover, abnormalities of the rest–activity circadian rhythm, including increased intra-daily variability (IV) and reduced inter-daily stability (IS) and relative amplitude of the rest–activity rhythm are described in AD (59, 60), and the presence of circadian dysfunction may predict a poor outcome in terms of cognitive functions (59).

We recently demonstrated the presence of variable degrees of rest–activity circadian dysfunction in mild–moderate AD patients and a specific loss of mRGCs in AD retinas (20). A specific AD pathology affecting these cells may contribute to circadian dysfunction in AD patients. Moreover, these cells have a direct effect on sleep through direct and indirect projections to brain nuclei relevant for sleep regulation such as the LH and the VLPO (61, 62). The role of mRGC loss in contributing to sleep and circadian misalignment in AD is particularly relevant for its potential therapeutic options. In fact, the use of bright light therapy has been proven to be effective in counteracting sleep and circadian dysfunction in AD (63, 64).

Other possible components of the circadian imbalance demonstrated in AD is the presence of SCN neuronal loss and amyloid pathology documented in neuropathological studies of AD postmortem brain (65, 66), which correlates with the degree of rest–activity disruption (67) and can contribute secondarily to the mRGC loss demonstrated in AD retinas.

The strict relationship between cognition, sleep, and circadian rhythms is highlighted also by the demonstration that the presence of circadian dysfunction may predict the onset of dementia (68), as well as that sleep loss may promote the accumulation of amyloid and predispose to AD (53, 69). Many recent studies point to direct and indirect effects of circadian derangement in cognitive disturbances and hence dementia. Counteracting the circadian imbalance may have important clinical implications. A summary of circadian abnormalities in AD is reported in Table 1.

**Table 1:** **Summary of circadian rhythm abnormalities in AD, PD, and HD**.

	Circadian rhythm abnormalities	Reference
AD	Daytime somnolence, increased sleep latency, and night-time awakenings Delayed phase of temperature circadian rhythm Sundowning Reduction of night-time melatonin levels Abnormal circadian expression profile of clock genes Increased IV, reduced IS, and reduced RA of rest–activity circadian rhythm	(3) (55) (56) (57) (57, 58) (20, 59, 60, 67)
PD	Abnormal melatonin circadian rhythm (phase advance and decreased amplitude) Increased IV, reduced IS, and reduced RA of rest–activity circadian rhythm Reversal of circadian BP rhythm and loss of HR variability Abnormal temperature and cortisol circadian rhythm Abnormal peripheral clock genes circadian rhythm	(70–73) (74–76) (77, 78) (73, 80) (73, 81)
HD	Delayed phase of the rest–activity rhythm Abnormal melatonin circadian rhythm Sleep fragmentation with night-time awakenings and reduced sleep efficiency	(88) (89) (90, 91)

*AD, Alzheimer’s disease; PD, Parkinson’s disease; HD, Huntington’s disease; IV, intra-daily variability; IS, inter-daily stability; RA, relative amplitude; BP, blood pressure; HR, heart rate*.

### Parkinson’s Disease

Sleep disturbances are reported in about 80% of PD patients (5). Furthermore, circadian dysfunction has been extensively described in PD patients, in terms of the following:

(1) Abnormal melatonin rhythm, i.e., abnormal phase angle of melatonin rhythm (70, 71) and decreased amplitude (72, 73). However, the phase advance of the melatonin rhythm documented by these studies was evident only in l-DOPA-treated PD patients, suggesting a possible influence of medications on these findings.
(2) Abnormal rest–activity rhythm, and in particular increased IV, reduced IS, and flattening of daily activity (74–76). However, a relevant influence of medications, motor, and non-motor symptoms (in particular cognitive disturbances and hallucinations) has been postulated also for these findings (6).
(3) Abnormal blood pressure (BP) and heart rate (HR) rhythm abnormalities such as reversal of circadian BP rhythm and loss of circadian HR variability (77, 78). However, these abnormalities can be influenced also by neurodegenerative changes of the autonomic nervous system documented in PD (79).
(4) Abnormal temperature (80) and cortisol rhythm (73).
(5) Abnormal clock gene rhythmicity in peripheral blood cells (73, 81).
(6) Circadian fluctuations of motor symptoms (82) with a worsening of motor functions possibly related to the dopamine level variations over the day.

Interestingly, at difference with AD, where neuropathological hallmarks of pathology such as amyloid plaques and neurofibrillary tangles are described in the SCN of the hypothalamus, the neurodegenerative changes characteristics of PD, such as synuclein deposition and Lewy bodies, are not reported in the SCN. This suggests that in PD, the circadian imbalance, at least in the early phase, is not primarily due to a master clock pathology (6). This is consistent with the finding that PD patients in the early stage of the disease do not exhibit frank circadian rhythm abnormalities, such as for melatonin and other hormones (6, 83). It is not clear, based on the currently available evidences, if circadian misalignment occurs as an independent hallmark of PD pathology or can be interpreted as a consequence of many other non-motor manifestations of PD, such as sleep, cognitive, and behavioral problems. Moreover, the investigation of circadian dysfunction in PD is hampered by the possible influence of many confounding factors, such as the motor fluctuations intrinsic to the disease and the influence of l-DOPA therapy. However, the presence of circadian imbalance in PD is well supported by circadian abnormalities described in many animal models of PD [for a review, see Ref. (79)].

In this complex scenario, a possible role in the pathogenesis of circadian problems described in PD patients can also involve the eye, and in particular the mRGC system. At this regard, there is documentation of a strict interaction between the mRGCs and the dopaminergic amacrine cells (84), a depletion of dopamine levels, and a specific synuclein deposition, particularly in the inner retina (39, 51, 52). Furthermore, a possible direct link between the eye, through the regulation of the melatonin synthesis, and the motor symptoms of PD has been postulated by Willis (85), as supported by the amelioration of motor symptoms after light exposure in PD patients (86). A summary of the main circadian abnormalities in PD is reported in Table 1.

In this wide scenario, it is possible that many factors, including the influence of mRGCs on modulating circadian rhythms and sleep, may play a role in the pathogenesis of circadian and sleep problems in PD. A more detailed investigation of this system is warranted, especially in *de novo* PD cases to elucidating the mechanisms behind.

## Beyond AD and PD: Huntington’s Disease

Sleep and circadian dysfunction occur early in the disease course of Huntington’s disease (HD) representing relevant non-motor symptoms of the disease [for a review, see Ref. (87)]. In particular, a delayed phase of rest–activity rhythm (88), an abnormal day–night ratio and melatonin rhythm (89), and consistent sleep fragmentation (90, 91) with increasing awakenings and reduced sleep efficiency have all been reported in HD.

Interestingly, the occurrence of sleep fragmentation and circadian misalignment in HD patients is relevant for aggravating the motor and cognitive problems of HD patients and bright light therapy improves motor and cognitive deficits in HD (90, 92). Moreover, even if there are evidences of neurodegenerative changes affecting the SCN in HD postmortem brain, the intact function of isolated SCN cells does not point to a primary central clock problem in the pathogenesis of circadian problems in HD, but more probably to a dysfunctional circuitry (87). Circadian abnormalities are also reported as early and prominent signs in the HD mouse models, the transgenic R6/2 and “knock-in” Q175 mice (93, 94). A summary of circadian abnormalities in HD is reported in Table 1.

Results on the possible occurrence of retinal degeneration in HD are contrasting, with some papers reporting the absence of retinal degeneration such as in the R6/2 mouse model (95) and others showing the presence of optic nerve degeneration (96, 97). In particular, a recent OCT study demonstrated the presence of temporal thinning in HD patients, which correlated with disease duration (97), with a pattern similar to PD and mitochondrial disorders (46, 47).

Interestingly, a recent study reported the occurrence of pupillary light response (PLR) dysfunction in R6/2 and Q175 mouse models, with a prevalent contribution of cone dysfunction in young–middle-aged mice and of mRGCs in old mice (98). In fact, a reduced PLR response is documented at low and moderate light intensity in young–middle-aged mice, whereas it is visible also at bright light in old mice, pointing to mRGC dysfunction. However, even if a significant reduction of melanopsin expression is evident in both mouse models also at early stages of the disease, the mRGCs are morphologically intact and do not show any signs of neurodegeneration. In particular, the aggregation of huntingtin protein is evident in a significant amount in the retina and in particular in the RGCs, but it is not recognized in the mRGCs except for the old animals, suggesting that mRGCs are relatively spared by neurodegeneration (98). These findings are in line with the findings in mitochondrial optic neuropathies, i.e., Leber’s hereditary optic neuropathy and dominant optic atrophy (DOA), where we demonstrated a relative resistance of mRGCs to mitochondrial dysfunction (99) and relative sparing of the PLR (100). This similarity can be explained by the significant contribution of mitochondrial dysfunction in HD pathogenesis (101, 102), including the mitochondrial dynamics alterations seen in HD, in particular increased mitochondrial fission (103), similar to DOA due to OPA1 mutations where fusion is affected (104).

However, even if the mRGCs are more resistant to neurodegenerative changes occurring in HD, the evidence of retinal pathology and, in particular, of reduced melanopsin expression in the retina of these mice can be relevant to the pathogenesis of circadian dysfunction in HD. These findings in HD are further examples that link the eye to the brain in a continuous dialog.

## Concluding Remarks

In this review, we summarized the recent findings of optic nerve pathology and its possible link with circadian dysfunction in AD (4, 20, 105, 106), PD (5, 44), and HD (87, 96–98) focusing in particular on the possible role of mRGCs in the pathogenesis of circadian dysfunction in these neurodegenerative disorders.

We also underscore the differences in the patterns of optic nerve degeneration described in these disorders, which predominantly affect the magnocellular RGCs of the retina in AD (16, 21) and the parvocellular RGCs in PD (44, 46, 49) and HD (97), possibly explained by the predominant mitochondrial dysfunction documented in PD and HD. Similarities and differences are also discussed in regards to the circadian rhythm imbalance documented in AD and PD.

The presence of neuropathological hallmarks, i.e., β-amyloid plaques (30, 107, 108), α-synuclein (51, 52), and huntingtin (98) in the retina of these neurodegenerative disorders demonstrates that the retina is specifically affected by neurodegeneration and affords access to potential biomarkers of the disease.

## Author Contributions

CLM was responsible for conception, design, drafting, and revision of the manuscript. FR-C, AS, and VC were responsible for conception and revision of the manuscript.

## Conflict of Interest Statement

The authors declare that the research was conducted in the absence of any commercial or financial relationships that could be construed as a potential conflict of interest.

## References

[1] Hickman RA Faustin A Wisniewski T. Alzheimer disease and its growing epidemic: risk factors, biomarkers, and the urgent need for therapeutics. Neurol Clin (2016) 34:941–53. 10.1016/j.ncl.2016.06.009 27720002 PMC5116320

[2] Lee A Gilbert RM. Epidemiology of Parkinson disease. Neurol Clin (2016) 34:955–65. 10.1016/j.ncl.2016.06.012 27720003

[3] Peter-Derex L Yammine P Bastuji H Croisile B Sleep and Alzheimer’s disease. Sleep Med Rev (2015) 19:29–38. 10.1016/j.smrv.2014.03.007 24846773

[4] Mattis J Sehgal A. Circadian rhythms, sleep, and disorders of aging. Trends Endocrinol Metab (2016) 27:192–203. 10.1016/j.tem.2016.02.003 26947521 PMC4808513

[5] Chahine LM Amara AW Videnovic A. A systematic review of the literature on disorders of sleep and wakefulness in Parkinson’s disease from 2005 to 2015. Sleep Med Rev (2016). 10.1016/j.smrv.2016.08.001 27863901 PMC5332351

[6] Fifel K Alterations of the circadian system in Parkinson’s disease patients. Mov Disord (2016). 10.1002/mds.26865 27859638

[7] Hattar S Liao HW Takao M Berson DM Yau KW. Melanopsin-containing retinal ganglion cells: architecture, projections, and intrinsic photosensitivity. Science (2002) 295:1065–70. 10.1126/science.1069609 11834834 PMC2885915

[8] Berson DM Dunn FA Takao M. Phototransduction by retinal ganglion cells that set the circadian clock. Science (2002) 295:1070–3. 10.1126/science.1067262 11834835

[9] Schmidt TM Do MT Dacey D Lucas R Hattar S Matynia A. Melanopsin-positive intrinsically photosensitive retinal ganglion cells: from form to function. J Neurosci (2011) 31:16094–101. 10.1523/JNEUROSCI.4132-11.2011 22072661 PMC3267581

[10] Schmoll C Lascaratos G Dhillon B Skene D Riha RL. The role of retinal regulation of sleep in health and disease. Sleep Med Rev (2011) 15:107–13. 10.1016/j.smrv.2010.06.001 21036633

[11] Münch M Kawasaki A. Intrinsically photosensitive retinal ganglion cells: classification, function and clinical implications. Curr Opin Neurol (2013) 26(1):45–51. 10.1097/WCO.0b013e32835c5e78 23254557

[12] Matynia A. Blurring the boundaries of vision: novel functions of intrinsically photosensitive retinal ganglion cells. J Exp Neurosci (2013) 7:43–50. 10.4137/JEN.S11267 25157207 PMC4089729

[13] Hinton DR Sadun AA Blanks JC Miller CA. Optic-nerve degeneration in Alzheimer’s disease. N Engl J Med (1986) 315:485–7. 10.1056/NEJM198608213150804 3736630

[14] Blanks JC Hinton DR Sadun AA Miller CA. Retinal ganglion cell degeneration in Alzheimer’s disease. Brain Res (1989) 501:364–72. 10.1016/0006-8993(89)90653-7 2819446

[15] Blanks JC Torigoe Y Hinton DR Blanks RH. Retinal degeneration in the macula of patients with Alzheimer’s disease. Ann N Y Acad Sci (1991) 640:44–6. 10.1111/j.1749-6632.1991.tb00188.x 1776758

[16] Sadun AA Bassi CJ. Optic nerve damage in Alzheimer’s disease. Ophthalmology (1990) 97:9–17. 10.1016/S0161-6420(90)32621-0 2314849

[17] Blanks JC Schmidt SY Torigoe Y Porrello KV Hinton DR Blanks RH. Retinal pathology in Alzheimer’s disease. II. Regional neuron loss and glial changes in GCL. Neurobiol Aging (1996) 17:385–95. 10.1016/0197-4580(96)00009-7 8725900

[18] Blanks JC Torigoe Y Hinton DR Blanks RH. Retinal pathology in Alzheimer’s disease. I. Ganglion cell loss in foveal/parafoveal retina. Neurobiol Aging (1996) 17:377–84. 10.1016/0197-4580(96)00010-3 8725899

[19] Curcio CA Drucker DN. Retinal ganglion cells in Alzheimer’s disease and aging. Ann Neurol (1993) 33:248–57. 10.1002/ana.410330305 8498808

[20] La Morgia C Ross-Cisneros FN Koronyo Y Hannibal J Gallassi R Cantalupo G Melanopsin retinal ganglion cell loss in Alzheimer disease. Ann Neurol (2016) 79:90–109. 10.1002/ana.24548 26505992 PMC4737313

[21] Coppola G Di Renzo A Ziccardi L Martelli F Fadda A Manni G Optical coherence tomography in Alzheimer’s disease: a meta-analysis. PLoS One (2015) 10:e0134750. 10.1371/journal.pone.0134750 26252902 PMC4529274

[22] Kesler A Vakhapova V Korczyn AD Naftaliev E Neudorfer M. Retinal thickness in patients with mild cognitive impairment and Alzheimer’s disease. Clin Neurol Neurosurg (2011) 113:523–6. 10.1016/j.clineuro.2011.02.014 21454010

[23] Kirbas S Turkyilmaz K Anlar O Tufekci A Durmus M. Retinal nerve fiber layer thickness in patients with Alzheimer disease. J Neuroophthalmol (2013) 33:58–61. 10.1097/WNO.0b013e318267fd5f 22918296

[24] Trick GL Trick LR Morris P Wolf M. Visual field loss in senile dementia of the Alzheimer’s type. Neurology (1995) 45:68–74. 10.1212/WNL.45.1.68 7824139

[25] Cheung CY Ong YT Hilal S Ikram MK Low S Ong YL Retinal ganglion cell analysis using high-definition optical coherence tomography in patients with mild cognitive impairment and Alzheimer’s disease. J Alzheimers Dis (2015) 45:45–56. 10.3233/JAD-141659 25428254

[26] Quigley HA Dunkelberger GR Green WR. Chronic human glaucoma causing selectively greater loss of large optic nerve fibers. Ophthalmology (1988) 95:357–63. 10.1016/S0161-6420(88)33176-3 3174003

[27] Cordeiro MF Migdal C Bloom P Fitzke FW Moss SE Imaging apoptosis in the eye. Eye (2011) 25:545–53. 10.1038/eye.2011.64 21436846 PMC3171262

[28] Koronyo-Hamaoui M Koronyo Y Ljubimov AV Miller CA Ko MK Black KL Identification of amyloid plaques in retinas from Alzheimer’s patients and noninvasive in vivo optical imaging of retinal plaques in a mouse model. Neuroimage (2011) 54(Suppl 1):S204–17. 10.1016/j.neuroimage.2010.06.020 20550967 PMC2991559

[29] Alexandrov PN Pogue A Bhattacharjee S Lukiw WJ. Retinal amyloid peptides and complement factor H in transgenic models of Alzheimer’s disease. Neuroreport (2011) 22:623–7. 10.1097/WNR.0b013e3283497334 21734608 PMC3719862

[30] Hart NJ Koronyo Y Black KL Koronyo-Hamaoui M. Ocular indicators of Alzheimer’s: exploring disease in the retina. Acta Neuropathol (2016) 132:767–87. 10.1007/s00401-016-1613-6 27645291 PMC5106496

[31] More SS Beach JM Vince R. Early detection of amyloidopathy in Alzheimer’s mice by hyperspectral endoscopy. Invest Ophthalmol Vis Sci (2016) 57:3231–8. 10.1167/iovs.15-17406 27333181

[32] Hamel M Emptage L Devries D Oliveros C Chow T Shah N Polarization properties of amyloid deposits in the retinas of an animal model of Alzheimer’s disease differ in those with and without cognitive impairment. Invest Ophthalmol Vis Sci ARVO Annual Meeting (2016) 57(12):2216.

[33] Pircher M Hitzenberger CK Schmidt-Erfurth U. Polarization sensitive optical coherence tomography in the human eye. Prog Retin Eye Res (2011) 30:431–51. 10.1016/j.preteyeres.2011.06.003 21729763 PMC3205186

[34] Schön C Hoffmann NA Ochs SM Burgold S Filser S Steinbach S Long-term in vivo imaging of fibrillar tau in the retina of P301S transgenic mice. PLoS One (2012) 7:e53547. 10.1371/journal.pone.0053547 23300938 PMC3534024

[35] Archibald NK Clarke MP Mosimann UP Burn DJ. The retina in Parkinson’s disease. Brain (2009) 132:1128–45. 10.1093/brain/awp068 19336464

[36] Weil RS Schrag AE Warren JD Crutch SJ Lees AJ Morris HR Visual dysfunction in Parkinson’s disease. Brain (2016) 139:2827–43. 10.1093/brain/aww175 PMC5091042 27412389

[37] Bodis-Wollner I. Visual deficits related to dopamine deficiency in experimental animals and Parkinson’s disease patients. Trends Neurosci (1990) 13:296–302. 10.1016/0166-2236(90)90113-O 1695407

[38] Bodis-Wollner I Electrophysiological assessment of retinal dopaminergic deficiency. Electroencephalogr Clin Neurophysiol Suppl (1996) 46:35–41. 9059777

[39] Harnois C Di Paolo T. Decreased dopamine in the retinas of patients with Parkinson’s disease. Invest Ophthalmol Vis Sci (1990) 31:2473–5. 2243012

[40] Hutton JT Morris JL Elias JW. Levodopa improves spatial contrast sensitivity in Parkinson’s disease. Arch Neurol (1993) 50:721–4. 10.1001/archneur.1993.00540070041012 8323475

[41] Silva MF Faria P Regateiro FS Forjaz V Januário C Freire A Independent patterns of damage within magno-, parvo- and koniocellular pathways in Parkinson’s disease. Brain (2005) 128:2260–71. 10.1093/brain/awh581 16000338

[42] Diederich NJ Pieri V Hipp G Rufra O Blyth S Vaillant M Discriminative power of different nonmotor signs in early Parkinson’s disease. A case-control study. Mov Disord (2010) 25:882–7. 10.1002/mds.22963 20198714

[43] Postuma RB Gagnon JF Vendette M Desjardins C Montplaisir JY. Olfaction and color vision identify impending neurodegeneration in rapid eye movement sleep behavior disorder. Ann Neurol (2011) 69:811–8. 10.1002/ana.22282 21246603

[44] Yu JG Feng YF Xiang Y Huang JH Savini G Parisi V Retinal nerve fiber layer thickness changes in Parkinson disease: a meta-analysis. PLoS One (2014) 9:e85718. 10.1371/journal.pone.0085718 24465663 PMC3897496

[45] Lee JY Ahn J Kim TW Jeon BS Optical coherence tomography in Parkinson’s disease: is the retina a biomarker? J Parkinsons Dis (2014) 4:197–204. 10.3233/JPD-130306 24518436

[46] La Morgia C Barboni P Rizzo G Carbonelli M Savini G Scaglione C Loss of temporal retinal nerve fibers in Parkinson disease: a mitochondrial pattern? Eur J Neurol (2013) 20:198–201. 10.1111/j.1468-1331.2012.03701.x 22436028

[47] Carelli V Ross-Cisneros FN Sadun AA. Mitochondrial dysfunction as a cause of optic neuropathies. Prog Retin Eye Res (2004) 23:53–89. 10.1016/j.preteyeres.2003.10.003 14766317

[48] Mendoza-Santiesteban CE Palma JA Martinez J Norcliffe-Kaufmann L Hedges TR III Kaufmann H. Progressive retinal structure abnormalities in multiple system atrophy. Mov Disord (2015) 30:1944–53. 10.1002/mds.26360 26359930 PMC4568758

[49] Bodis-Wollner I Miri S Glazman S. Venturing into the no-man’s land of the retina in Parkinson’s disease. Mov Disord (2014) 29:15–22. 10.1002/mds.25741 24339212

[50] Shrier EM Adam CR Spund B Glazman S Bodis-Wollner I. Interocular asymmetry of foveal thickness in Parkinson disease. J Ophthalmol (2012) 2012:728457. 10.1155/2012/728457 22900149 PMC3415246

[51] Bodis-Wollner I Kozlowski PB Glazman S Miri S. α-Synuclein in the inner retina in Parkinson disease. Ann Neurol (2014) 75:964–6. 10.1002/ana.24182 24816946

[52] Beach TG Carew J Serrano G Adler CH Shill HA Sue LI Phosphorylated α-synuclein-immunoreactive retinal neuronal elements in Parkinson’s disease subjects. Neurosci Lett (2014) 571:34–8. 10.1016/j.neulet.2014.04.027 24785101 PMC4591751

[53] Musiek ES Holtzman DM. Mechanisms linking circadian clocks, sleep, and neurodegeneration. Science (2016) 354:1004–8. 10.1126/science.aah4968 27885006 PMC5219881

[54] Petit D Gagnon JF Fantini ML Ferini-Strambi L Montplaisir J Sleep and quantitative EEG in neurodegenerative disorders. J Psychosom Res (2004) 56:487–96. 10.1016/j.jpsychores.2004.02.001 15172204

[55] Satlin A Volicer L Stopa EG Harper D. Circadian locomotor activity and core-body temperature rhythms in Alzheimer’s disease. Neurobiol Aging (1995) 16:765–71. 10.1016/0197-4580(95)00059-N 8532109

[56] Volicer L Harper DG Manning BC Goldstein R Satlin A. Sundowning and circadian rhythms in Alzheimer’s disease. Am J Psychiatry (2001) 158:704–11. 10.1176/appi.ajp.158.5.704 11329390

[57] Weissová K Bartoš A Sládek M Nováková M Sumová A. Moderate changes in the circadian system of Alzheimer’s disease patients detected in their home environment. PLoS One (2016) 11:e0146200. 10.1371/journal.pone.0146200 26727258 PMC4701009

[58] Cermakian N Lamont EW Boudreau P Boivin DB. Circadian clock gene expression in brain regions of Alzheimer’s disease patients and control subjects. J Biol Rhythms (2011) 26:160–70. 10.1177/0748730410395732 21454296

[59] Hatfield CF Herbert J van Someren EJ Hodges JR Hastings MH. Disrupted daily activity/rest cycles in relation to daily cortisol rhythms of home-dwelling patients with early Alzheimer’s dementia. Brain (2004) 127:1061–74. 10.1093/brain/awh129 14998915

[60] Hooghiemstra AM Eggermont LH Scheltens P van der Flier WM Scherder EJ. The rest-activity rhythm and physical activity in early-onset dementia. Alzheimer Dis Assoc Disord (2015) 29:45–9. 10.1097/WAD.0000000000000037 24632989

[61] Lupi D Oster H Thompson S Foster RG. The acute light-induction of sleep is mediated by OPN4-based photoreception. Nat Neurosci (2008) 11:1068–73. 10.1038/nn.2179 19160505

[62] Pilorz V Tam SK Hughes S Pothecary CA Jagannath A Hankins MW Melanopsin regulates both sleep-promoting and arousal-promoting responses to light. PLoS Biol (2016) 14:e1002482 10.1371/journal.pbio.1002482 27276063 PMC4898879

[63] Figueiro MG Plitnick BA Lok A Jones GE Higgins P Hornick TR Tailored lighting intervention improves measures of sleep, depression, and agitation in persons with Alzheimer’s disease and related dementia living in long-term care facilities. Clin Interv Aging (2014) 9:1527–37. 10.2147/CIA.S68557 25246779 PMC4168854

[64] Hanford N Figueiro M. Light therapy and Alzheimer’s disease and related dementia: past, present, and future. J Alzheimers Dis (2013) 33:913–22. 10.3233/JAD-2012-121645 23099814 PMC3553247

[65] Stopa EG Volicer L Kuo-Leblanc V Harper D Lathi D Tate B Pathologic evaluation of the human suprachiasmatic nucleus in severe dementia. J Neuropathol Exp Neurol (1999) 58:29–39. 10.1097/00005072-199901000-00004 10068311

[66] Harper DG Stopa EG Kuo-Leblanc V McKee AC Asayama K Volicer L Dorsomedial SCN neuronal subpopulations subserve different functions in human dementia. Brain (2008) 131:1609–17. 10.1093/brain/awn049 18372313 PMC3286014

[67] Wang JL Lim AS Chiang WY Hsieh WH Lo MT Schneider JA Suprachiasmatic neuron numbers and rest-activity circadian rhythms in older humans. Ann Neurol (2015) 78:317–22. 10.1002/ana.24432 25921596 PMC4515161

[68] Tranah GJ Blackwell T Stone KL Ancoli-Israel S Paudel ML Ensrud KE Circadian activity rhythms and risk of incident dementia and mild cognitive impairment in older women. Ann Neurol (2011) 70:722–32. 10.1002/ana.22468 22162057 PMC3244839

[69] Kang JE Lim MM Bateman RJ Lee JJ Smyth LP Cirrito JR Amyloid-beta dynamics are regulated by orexin and the sleep-wake cycle. Science (2009) 326:1005–7. 10.1126/science.1180962 19779148 PMC2789838

[70] Fertl E Auff E Doppelbauer A Waldhauser F. Circadian secretion pattern of melatonin in de novo parkinsonian patients: evidence for phase-shifting properties of l-DOPA. J Neural Transm Park Dis Dement Sect (1993) 5:227–34. 10.1007/BF02257677 8369102

[71] Bolitho SJ Naismith SL Rajaratnam SM Grunstein RR Hodges JR Terpening Z Disturbances in melatonin secretion and circadian sleep-wake regulation in Parkinson disease. Sleep Med (2014) 15:342–7. 10.1016/j.sleep.2013.10.016 24529544

[72] Videnovic A Noble C Reid KJ Peng J Turek FW Marconi A Circadian melatonin rhythm and excessive daytime sleepiness in Parkinson disease. JAMA Neurol (2014) 71:463–9. 10.1001/jamaneurol.2013.6239 24566763 PMC4078989

[73] Breen DP Vuono R Nawarathna U Fisher K Shneerson JM Reddy AB Sleep and circadian rhythm regulation in early Parkinson disease. JAMA Neurol (2014) 71:589–95. 10.1001/jamaneurol.2014.65 24687146 PMC4119609

[74] Van Hilten JJ Hoogland G van der Velde EA van Dijk JG Kerkhof GA Roos RA. Quantitative assessment of parkinsonian patients by continuous wrist activity monitoring. Clin Neuropharmacol (1993) 16:36–45. 10.1097/00002826-199302000-00004 8422656

[75] Whitehead DL Davies AD Playfer JR Turnbull CJ. Circadian rest-activity rhythm is altered in Parkinson’s disease patients with hallucinations. Mov Disord (2008) 23:1137–45. 10.1002/mds.2205 18442142

[76] Niwa F Kuriyama N Nakagawa M Imanishi J. Circadian rhythm of rest activity and autonomic nervous system activity at different stages in Parkinson’s disease. Auton Neurosci (2011) 165:195–200. 10.1016/j.autneu.2011.07.010 21871844

[77] Ejaz AA Sekhon IS Munjal S. Characteristic findings on 24-h ambulatory blood pressure monitoring in a series of patients with Parkinson’s disease. Eur J Intern Med (2006) 17(6):417–20. 10.1016/j.ejim.2006.02.020 16962949

[78] Devos D Kroumova M Bordet R Vodougnon H Guieu JD Libersa C Heart rate variability and Parkinson’s disease severity. J Neural Transm (Vienna) (2003) 110:997–1011. 10.1007/s00702-003-0016-8 12928836

[79] Videnovic A Willis GL Circadian system – a novel diagnostic and therapeutic target in Parkinson’s disease? Mov Disord (2016) 3:260–9. 10.1002/mds.26509 PMC4783245 26826022

[80] Zhong G Bolitho S Grunstein R Naismith SL Lewis SJ. The relationship between thermoregulation and REM sleep behaviour disorder in Parkinson’s disease. PLoS One (2013) 8(8):e72661. 10.1371/journal.pone.0072661 23991135 PMC3749164

[81] Cai Y Liu S Sothern RB Xu S Chan P. Expression of clock genes Per1 and Bmal1 in total leukocytes in health and Parkinson’s disease. Eur J Neurol (2010) 17:550–4. 10.1111/j.1468-1331.2009.02848.x 19912323

[82] Bonuccelli U Del Dotto P Lucetti C Petrozzi L Bernardini S Gambaccini G Diurnal motor variations to repeated doses of levodopa in Parkinson’s disease. Clin Neuropharmacol (2000) 23:28–33. 10.1097/00002826-200001000-00006 10682228

[83] Aziz NA Pijl H Frölich M Roelfsema F Roos RA Leptin, adiponectin, and resistin secretion and diurnal rhythmicity are unaltered in Parkinson’s disease. Mov Disord (2011) 26:760–1. 10.1002/mds.23463 21271615

[84] Zhang DQ Wong KY Sollars PJ Berson DM Pickard GE McMahon DG. Intraretinal signaling by ganglion cell photoreceptors to dopaminergic amacrine neurons. Proc Natl Acad Sci U S A (2008) 105:14181–6. 10.1073/pnas.0803893105 18779590 PMC2544598

[85] Willis GL. Parkinson’s disease as a neuroendocrine disorder of circadian function: dopamine-melatonin imbalance and the visual system in the genesis and progression of the degenerative process. Rev Neurosci (2008) 19:245–316. 10.1515/REVNEURO.2008.19.4-5.245 19145986

[86] Willis GL Moore C Armstrong SM. A historical justification for and retrospective analysis of the systematic application of light therapy in Parkinson’s disease. Rev Neurosci (2012) 23:199–226. 10.1515/revneuro-2011-0072 22499678

[87] Morton AJ. Circadian and sleep disorder in Huntington’s disease. Exp Neurol (2013) 243:34–44. 10.1016/j.expneurol.2012.10.014 23099415

[88] Aziz NA Anguelova GV Marinus J Lammers GJ Roos RA. Sleep and circadian rhythm alterations correlate with depression and cognitive impairment in Huntington’s disease. Parkinsonism Relat Disord (2010) 16:345–50. 10.1016/j.parkreldis.2010.02.009 20236854

[89] Aziz NA Pijl H Frölich M Schröder-van der Elst JP van der Bent C Roelfsema F Delayed onset of the diurnal melatonin rise in patients with Huntington’s disease. J Neurol (2009) 256:1961–5. 10.1007/s00415-009-5196-1 19562249 PMC2780627

[90] Morton AJ Wood NI Hastings MH Hurelbrink C Barker RA Maywood ES. Disintegration of the sleep-wake cycle and circadian timing in Huntington’s disease. J Neurosci (2005) 25:157–63. Erratum in: *J Neurosci* (2005) 25(15):3994. 10.1523/JNEUROSCI.3842-04.2005 15634777 PMC6725210

[91] Goodman AO Rogers L Pilsworth S McAllister CJ Shneerson JM Morton AJ Asymptomatic sleep abnormalities are a common early feature in patients with Huntington’s disease. Curr Neurol Neurosci Rep (2011) 11:211–7. 10.1007/s11910-010-0163-x 21103960

[92] Pallier PN Morton AJ. Management of sleep/wake cycles improves cognitive function in a transgenic mouse model of Huntington’s disease. Brain Res (2009) 1279:90–8. 10.1016/j.brainres.2009.03.072 19450569

[93] Kudo T Schroeder A Loh DH Kuljis D Jordan MC Roos KP Dysfunctions in circadian behavior and physiology in mouse models of Huntington’s disease. Exp Neurol (2011) 228:80–90. 10.1016/j.expneurol.2010.12.011 21184755 PMC4346330

[94] Loh DH Kudo T Truong D Wu Y Colwell CS. The Q175 mouse model of Huntington’s disease shows gene dosage- and age-related decline in circadian rhythms of activity and sleep. PLoS One (2013) 8:e69993. 10.1371/journal.pone.0069993 23936129 PMC3728350

[95] Ragauskas S Leinonen H Puranen J Rönkkö S Nymark S Gurevicius K Early retinal function deficit without prominent morphological changes in the R6/2 mouse model of Huntington’s disease. PLoS One (2014) 9:e113317 10.1371/journal.pone.0113317 25469887 PMC4254453

[96] Andrade C Beato J Monteiro A Costa A Penas S Guimarães J Spectral-domain optical coherence tomography as a potential biomarker in Huntington’s disease. Mov Disord (2016) 31:377–83. 10.1002/mds.26486 26853218

[97] Kersten HM Danesh-Meyer HV Kilfoyle DH Roxburgh RH. Optical coherence tomography findings in Huntington’s disease: a potential biomarker of disease progression. J Neurol (2015) 262:2457–65. 10.1007/s00415-015-7869-2 26233693

[98] Ouk K Hughes S Pothecary CA Peirson SN Jennifer Morton A Attenuated pupillary light responses and downregulation of opsin expression parallel decline in circadian disruption in two different mouse models of Huntington’s disease. Hum Mol Genet (2016) 25:5418–32. 10.1093/hmg/ddw359 PMC5418835 28031289

[99] La Morgia C Ross-Cisneros FN Sadun AA Hannibal J Munarini A Mantovani V Melanopsin retinal ganglion cells are resistant to neurodegeneration in mitochondrial optic neuropathies. Brain (2010) 133:2426–38. 10.1093/brain/awq155 20659957 PMC3139936

[100] Moura AL Nagy BV La Morgia C Barboni P Oliveira AG Salomão SR The pupil light reflex in Leber’s hereditary optic neuropathy: evidence for preservation of melanopsin-expressing retinal ganglion cells. Invest Ophthalmol Vis Sci (2013) 54:4471–7. 10.1167/iovs.12-11137 23737476 PMC4322722

[101] Guedes-Dias P Pinho BR Soares TR de Proença J Duchen MR Oliveira JM. Mitochondrial dynamics and quality control in Huntington’s disease. Neurobiol Dis (2016) 90:51–7. 10.1016/j.nbd.2015.09.008 26388396

[102] Oliveira JM. Nature and cause of mitochondrial dysfunction in Huntington’s disease: focusing on huntingtin and the striatum. J Neurochem (2010) 114:1–12. 10.1111/j.1471-4159.2010.06741.x 20403078

[103] Song W Chen J Petrilli A Liot G Klinglmayr E Zhou Y Mutant huntingtin binds the mitochondrial fission GTPase dynamin-related protein-1 and increases its enzymatic activity. Nat Med (2011) 17:377–82. 10.1038/nm.2313 21336284 PMC3051025

[104] Yu-Wai-Man P Votruba M Burté F La Morgia C Barboni P Carelli V. A neurodegenerative perspective on mitochondrial optic neuropathies. Acta Neuropathol (2016) 132:789–806. 10.1007/s00401-016-1625-2 27696015 PMC5106504

[105] Lim JK Li QX He Z Vingrys AJ Wong VH Currier N The eye as a biomarker for Alzheimer’s disease. Front Neurosci (2016) 10:536. 10.3389/fnins.2016.00536 27909396 PMC5112261

[106] Javaid FZ Brenton J Guo L Cordeiro MF. Visual and ocular manifestations of Alzheimer’s disease and their use as biomarkers for diagnosis and progression. Front Neurol (2016) 7:55. 10.3389/fneur.2016.00055 27148157 PMC4836138

[107] Masuzzo A Dinet V Cavanagh C Mascarelli F Krantic S. Amyloidosis in retinal neurodegenerative diseases. Front Neurol (2016) 7:127. 10.3389/fneur.2016.00127 27551275 PMC4976396

[108] Shah TM Gupta SM Chatterjee P Campbell M Martins RN. Beta-amyloid sequelae in the eye: a critical review on its diagnostic significance and clinical relevance in Alzheimer’s disease. Mol Psychiatry (2017) 22(3):353–63. 10.1038/mp.2016.251 28093567

